# Inhibition of the activity of pro-inflammatory secretory phospholipase A_2_ by acute phase proteins

**DOI:** 10.1155/S0962935196000270

**Published:** 1996-06

**Authors:** W. Pruzanski, E. Stefanski, P. Vadas

**Affiliations:** 1 Inflammation Research Group The Wellesley Hospital Research Institute University of Toronto Ontario M4Y 1J3 Canada

## Abstract

Pro-Inflammatory non-pancreatic phospholipase A_2_ (sPLA_2_) is markedly over-expressed in acute systemic and chronic local inflammatory processes. Since in acute phase reaction sPLA_2_ is often over-expressed simultaneously with acute phase proteins (APP), it is important to determine whether APP interacts with sPLA_2_. We tested ten APPs for interaction with sPLA_2_ using as a substrate multilamellar Hposomes composed either of PC:Lyso PC or PE:Lyso PE. Using PC:Lyso PC substrate, CRP, lactoferrin and SAP were found to inhibit sPLA_2_ activity with an IC_50_ of 25 μg/ml, 7.5 μg/ml and 50 μg/ml, respectively, corresponding to 0.21 μM, 0.1 μM and 0.21 μM respectively. Using PE:Lyso PE substrate only SAP was inhibitory, with an IC_50_ of 10 μg/ml (0.04 μM). Phosphorylcholine abolished the inhibitory activity of CRP but not of SAP or lactoferrin. Addition of phosphorylethanolamine or of excess calcium had no effect on the inhibitory activity of APP. Limulin, lysozyme, transferrin, β_2_-microglobulin, α_2_-macroglobulin, human and bovine albumins had no effect on sPLA2 activity. Therefore neither the structure of pentraxins, or ironbinding, bacteriostatic property or amyloidogenic property preclude whether APP modulates sPLA_2_ activity. Inhibition of pro-inflammatory sPLA_2_ by APP may be one of the protective mechanisms of the acute phase reaction.


Mediators of Inflammation, 5, 196–201 (1996)

### Inhibition of the activity of pro-inflammatory secretory phospholipase $A_2$ by acute phase proteins

W. Pruzanski,[CA] E. Stefanski and P. Vadas

Inflammation Research Group, The Wellesley Hospital Research Institute, University of Toronto, Ontario, Canada, M4Y 1J3

[CA]Corresponding Author

**Pro-inflammatory non-pancreatic phospholipase $A_2$ ($sPLA_2$) is markedly over-expressed in acute systemic and chronic local inflammatory processes. Since in acute phase reaction $sPLA_2$ is often over-expressed simultaneously with acute phase proteins (APP), it is important to determine whether APP interacts with $sPLA_2$. We tested ten APPs for interaction with $sPLA_2$ using as a substrate multilamellar liposomes composed either of PC:Lyso PC or PE:Lyso PE. Using PC:Lyso PC substrate, CRP, lactoferrin and SAP were found to inhibit $sPLA_2$ activity with an $IC_{50}$ of 25 μg/ml, 7.5 μg/ml and 50 μg/ml, respectively, corresponding to 0.21 μM, 0.1 μM and 0.21 μM respectively. Using PE:Lyso PE substrate only SAP was inhibitory, with an $IC_{50}$ of 10 μg/ml (0.04 μM). Phosphorylcholine abolished the inhibitory activity of CRP but not of SAP or lactoferrin. Addition of phosphorylethanolamine or of excess calcium had no effect on the inhibitory activity of APP. Limulin, lysozyme, transferrin, $\beta_2$-microglobulin, $\alpha_2$-macroglobulin, human and bovine albumins had no effect on $sPLA_2$ activity. Therefore neither the structure of pentraxins, or iron-binding, bacteriostatic property or amyloidogenic property preclude whether APP modulates $sPLA_2$ activity. Inhibition of pro-inflammatory $sPLA_2$ by APP may be one of the protective mechanisms of the acute phase reaction.**

**Key words:** Acute phase proteins, Inflammation, Phospholipase $A_2$

### Introduction

Secretory non-pancreatic phospholipase $A_2$ ($sPLA_2$) belongs to the group of low-molecular-weight, calcium-dependent, lipolytic enzymes.[1] It plays an important physiological role in host defence participating in the destruction of Gram-negative microorganisms.[2,3] sPLA was also found to exert modulatory activity on the cellular proliferation and tumour formation in the intestinal tract.[4,5] Excessive activity of circulating $sPLA_2$ was discovered in several systemic inflammatory response syndromes (SIRS) such as clinical and experimental sepsis, multiorgan failure and salicylate intoxication,[6–9] and in more localized processes such as peritonitis and inflammatory arthritis.[10,11]A pathogenetic role of $sPLA_2$ was implicated by the observation that enzymatic activity and immunoreactivity of $sPLA_2$ in SIRS correlated with the severity and outcome of the disease,[6–9] and by the fact that hypotension induced either by Gram-negative microorganisms[7] or by infusion of $sPLA_2$[6] could be attenuated by inhibition of the enzyme. Acute inflammatory processes induced by $sPLA_2$ administered intracutaneously,[12] into subcutaneous air pouches[13] or intraarticularly,[14,15] could be blocked by inhibitors of $PLA_2$.[12–15]

In general terms inhibition of $sPLA_2$ can be induced by three mechanisms: inhibition of synthesis, competition for substrate, or direct inhibition of $sPLA_2$ enzymatic activity. The best characterized inducers of its synthesis are cytokines, such as IL-1 and TNF.[16] IL-6 and oncostatin M induce $sPLA_2$ in cells of hepatic origin such as Hep $G_2$[17,18] and normal human liver cells (W. Pruzanski *et al.*, unpublished). Endotoxin was also found to be a strong inducer of $sPLA_2$.[18] The above observations were consistent with the postulate that $sPLA_2$ plays an important pathogenetic role in inflammatory processes and led to an extensive search for exogenous inhibitors. However, very little is known about endogenous modulation of $sPLA_2$. Two endogenous inhibitors, glucocorticoid-inducible lipocortin[19] and complement fragments[20] have been found, but neither has evolved into a useful therapeutic agent.

© 1996 Rapid Science Publishers

During the acute phase reaction the synthesis and release of $sPLA_2$ occurs simultaneously with that of a large number of acute phase proteins.[21,22] Experimental studies have shown that endotoxin, IL-1, TNF and IL-6 are the main inducers of such release.[21,22] Therefore the process of induction of acute phase proteins by liver cells seems to be similar to that of $sPLA_2$. Little is known about the possible interactions of acute phase proteins. We reported recently that CRP, one of the classical acute phase proteins is a strong inhibitor of $sPLA_2$, binding competitively to the phosphorylcholine-containing substrates[23] whereas SAA, another acute phase protein, enhances $sPLA_2$ activity.[24] Herein we report that two other acute phase proteins, serum amyloid P (SAP) and lactoferrin suppress $sPLA_2$ activity. These, previously unrecognized, interactions of acute phase proteins with proinflammatory $sPLA_2$, may add a new aspect to the understanding of the complex role of acute phase reaction.

### Materials and Methods

1,2-Dipalmitoyl-phosphatidylcholine (dipalmitoyl PC) was obtained from Avanti Polar Lipids (Birmingham, AL). Phosphatidylcholine L-α-dipalmitoyl [2-palmitoyl-1-$^{14}C$] (55.5 mCi/mmol) and oleic acid [1-$^{14}C$] (40–60 mCi/mmol) were purchased from DuPont NEN Products. L-3-phosphatidylethanolamine 1-palmitoyl [2-$^{14}C$ linoleoyl] (50–60 mCi/mmol) was obtained from Amersham (Arlington Heights, IL). Recombinant human $sPLA_2$ (rh-$sPLA_2$) was a generous gift of Dr Jeffrey Browning, Biogen Corporation (Cambridge, MA). Bio-Rad protein assay reagent was purchased from Bio-Rad (Richmond, CA). L-α-lysophosphatidylcholine palmitoyl, L-α-phosphatidylethanolamine-β-linoleoyl-γ-palmitoyl, L-α-lysophosphatidylethanolamine palmitoyl, bovine serum albumin and silica gel were purchased from Sigma Chemical Corporation. All reagents were analytical grade or better.

Recombinant human lysozyme, bovine serum albumin (BSA), human serum albumin (HSA), lactoferrin purified from human milk, transferrin, $\beta_2$-microglobulin purified from human urine, limulin from *Limulus polyphemus* and $\alpha_2$-macroglobulin were obtained from Sigma Chemical Company (St Louis, MO). CRP purified from human plasma was obtained from Helix Biotech Corporation, Richmond, BC and SAP purified from human serum, from Calbiochem, San Diego, CA.

*Liposome assay:* Aliquots of dipalmitoyl PC, [$^{14}C$] dipalmitoyl PC, with or without LysoPC (2:1) or phosphatidylethanolamine (PE) with or without LysoPE (2:1) were prepared in chloroform and evaporated to dryness. Multilamellar liposomes were made by dispersing the resulting lipid mixture in 100 mM Tris HCl buffer, pH 8.0, followed by heating for 2 min at 41°C and vortexing for 2 min before use. Only freshly prepared liposomes were used. Assays were carried out in a total volume of 0.2 ml of 100 mM Tris HCl, pH 8.0 containing 2.5 or 10 mM $CaCl_2$, 0.1% bovine serum albumin and 5–30 nmoles of PC (or PE) vesicles (containing 2–16 nCi of [$^{14}C$] dipalmitoyl PC per assay). The optimal concentration was found to be 20 nmoles/assay and this was used in all experiments. If acute phase proteins were included, they were preincubated with 20 nmoles of liposomes for 1 h at 41°C before the assay. The reaction was then started by addition of 20 μl of recombinant human $sPLA_2$ stock solution with final $sPLA_2$ concentrations ranging between 10 and 200 ng/200 μl assay volume unless otherwise stated. The reaction mixture was then incubated for 30 min at 41°C. The reaction was stopped by the addition of 1.32 ml isopropanol/heptane/0.5 M $H_2SO_4$ 40:10:1 (v/v/v). The mixture was heated for 1 min at 60°C before addition of 0.66 ml $H_2O$ and 0.8 ml heptane. The two phases were allowed to separate and after centrifugation for 10 min at 1 500 rpm, 0.8 ml of the upper phase was added to 1.0 ml heptane containing 100 mg silica gel. The mixture was spun again for 10 min at 1 500 rpm and 1.0 ml of the supernatant was used for scintillation counting of $^{14}C$-labelled free palmitic acid. All assays were done in triplicate.

*Immunologic assays:* Anti-human (mouse IgG2) CRP/SAP antibody (clone CRP-20) C6552 was obtained from Sigma (St Louis, MO). It reacted with an epitope located on the 24 kDa subunit of denatured and reduced CRP and it recognized CRP independently of the $Ca^{2+}$ binding site. This antibody did not recognize the calcium dependent phosphorylcholine binding site of CRP. It cross-reacted with human SAP, but not with CRP from *Limulus polyphemus*. Working dilutions were at least 1:4 000 per μg of antigen in indirect ELISA assay. In antibody assays, the antigens were preincubated with appropriately diluted antibodies for 60 min at room temperature. Then the liposomal substrate was added and the mixture was further incubated for 60 min at 41°C. The $PLA_2$ was finally added and the incubation was carried on for an additional 30 min at 41°C.

Each experiment was repeated at least three times. The differences between the results and controls were assessed by Student's *t*-test.

### Results

Ten acute phase proteins were tested for modulation of $sPLA_2$ activity, using as a substrate multilamellar liposomes composed either of PC:Lyso PC or PE:Lyso PE in the ratio of 2:1. Using the former substrate, lactoferrin (Fig. 1) CRP (Fig. 2) and SAP (Fig. 3) were inhibitory, with an $IC_{50}$ of 7.5 μg/ml, 25 μg/ml and 50 μg/ml respectively. These concentrations corresponded to 0.10 μM, 0.21 μM and 0.21 μM respectively (Fig. 4). Using the latter substrate, only SAP was inhibitory, with an $IC_{50}$ of 10 μg/ml (0.04 μM) (Fig. 3). Using mixed substrate (PE:Lyso PC), no inhibition of $sPLA_2$ activity by CRP was noted (Table 1).

Addition of phosphorylcholine in concentrations ranging from 0.5 to 10 μM abolished inhibitory activity of CRP. Fifty per cent attenuation was achieved by 2.0 μM and complete inhibition by 10 μM of phosphorylcholine.[23] Phosphorylcholine did not alter inhibitory activity of SAP or of lactoferrin. Phosphorylethanolamine, 10 μM,

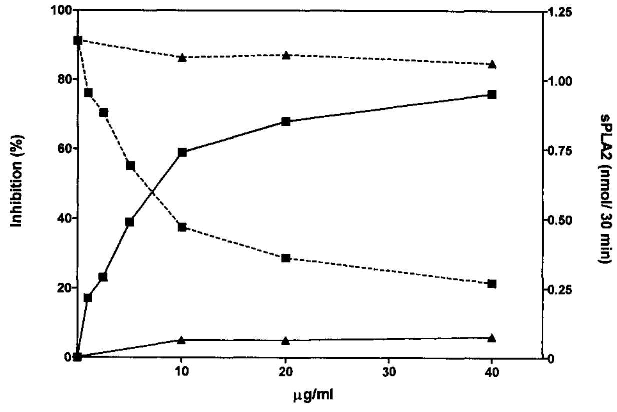

FIG. 1. Inhibition of $sPLA_2$ activity by lactoferrin. Closed squares, solid line—inhibition of $sPLA_2$ activity using PC:Lyso PC multilamellar liposomes as a substrate. Closed squares, interrupted line—as above; expression of $sPLA_2$ in nmol/30 min. Closed triangles, solid line—inhibition of $sPLA_2$ activity using PE:Lyso PE multilamellar liposomes as a substrate. Closed triangles, interrupted line—as above; expresssion of $sPLA_2$ in nmoles/30 min.

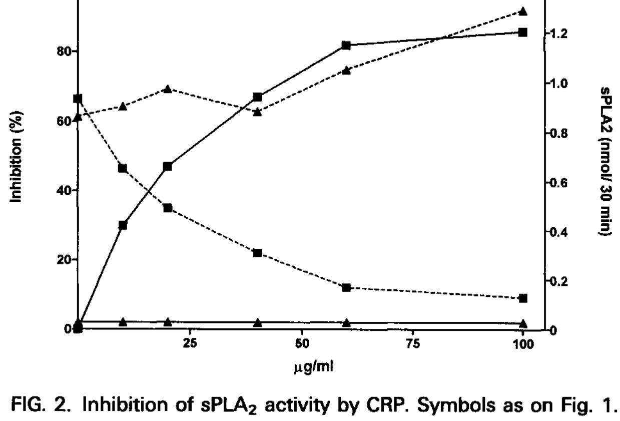

FIG. 2. Inhibition of $sPLA_2$ activity by CRP. Symbols as on Fig. 1.

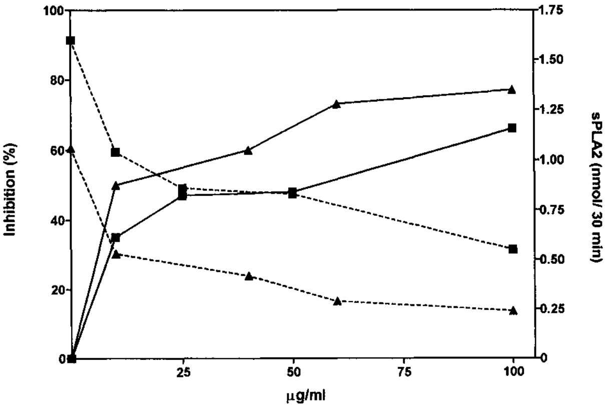

FIG. 3. Inhibition of $sPLA_2$ activity by SAP. Symbols as on Fig. 1.

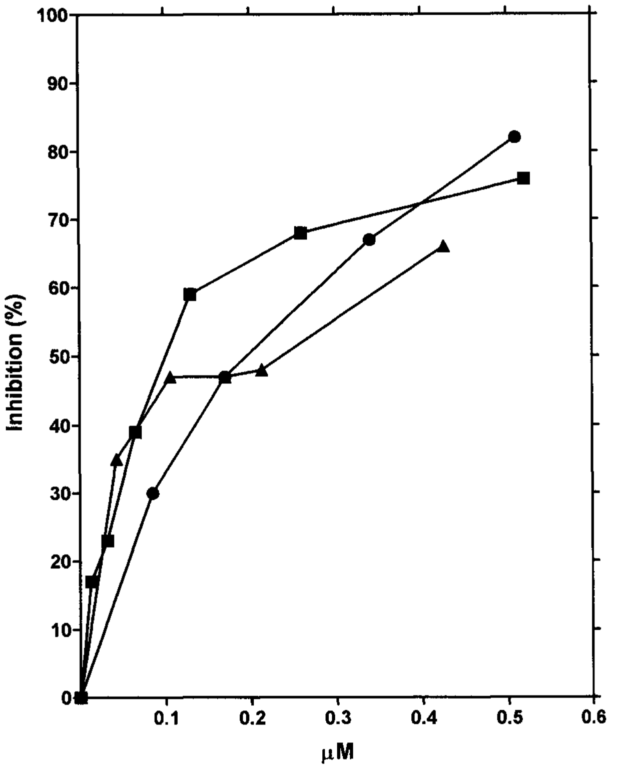

FIG. 4. Comparison of inhibitory activity of lactoferrin (squares), SAP (triangles) and CRP (circles) against $sPLA_2$, expressed in micromolar concentrations. PC:Lyso PC multilamellar liposomes used as a substrate.

had no effect on inhibitory activity of SAP, CRP or lactoferrin.

Anti CRP/SAP monoclonal antibody alone had no effect on $sPLA_2$ activity. This antibody preincubated with CRP or SAP did not alter their inhibitory activity in the range of concentrations used in the assays (CRP or SAP 40 μg/ml, $sPLA_2$ 100 ng).

To test whether chelation may have an effect on activity of $sPLA_2$, various concentrations of calcium were tested using either PC:Lyso PC or

**Table 1.** Impact of substrate on inhibitory activity of CRP

| Substrate | $sPLA_2$ (nmoles/30') |
|---|---|
| PE:Lyso PE | 0.99 |
| PE:Lyso PE + CRP | 1.09 |
| PC:Lyso PC | 0.93 |
| PC:Lyso PC + CRP | 0.31 |
| PE:Lyso PC | 0.93 |
| PE:Lyso PC + CRP | 1.35 |

*$sPLA_2$ 100 ng, CRP 40 μg/ml.

PE:Lyso PE as a substrate. Maximum activity of $sPLA_2$ was observed at the level of 0.5–1.0 μM $Ca^{2+}$. In some experiments with CRP, SAP, or lactoferrin, $Ca^{2+}$ concentrations were increased up to 20 μM. Excess of $Ca^{2+}$ had no effect on inhibitory activity of these proteins.

Human and bovine serum albumin, transferrin, lysozyme, $\beta_2$-microglobulin, $\alpha_2$-macroglobulin and limulin had no effect on the activity of $sPLA_2$.

### Discussion

An insult to an organism's homeostasis caused by injury, infection or inflammation, leads to a swift systemic response called the acute phase reaction (APR) (reviewed in References 21, 25–27). In the case of chronic or recurrent inflammation, APR may become quite prolonged.[25] An integral part of APR is a rapid synthesis and extracellular release of a large number of acute phase proteins (APP), and simultaneous decrease in some other proteins, the so called negative APP. Liver is the major source of APP synthesis, but other cells participate in the synthesis of APP as well.[22,26,28] APR is orchestrated by a group of inflammatory mediators, including glucocorticoids, cytokines, anaphylotoxins and growth factors.[21,25] The group of cytokines which induce APP synthesis includes, but is not limited to, IL-1, TNF, IL-6 and oncostatin.[21,22,26,29,30]

The same group of cytokines was also found to induce the synthesis and release of the pro-inflammatory enzyme, secretory non-pancreatic phospholipase $A_2$ ($sPLA_2$).[16,18] $sPLA_2$, first discovered in experimental peritonitis[31] and in fluids draining inflammatory sites,[32] was found to raise rapidly in the circulation in systemic inflammatory response syndromes such as septic shock,[7] salicylate poisoning[9] and malaria[33] and in the milieu of more localized inflammatory sites such as arthritis.[11] In the former group, circulating $sPLA_2$ correlated with both complications and the outcome of the disease,[7] whereas in the latter it correlated with the disease activity.[11]

Since the increase in circulatory $sPLA_2$ activity parallels temporally that of acute phase proteins, it was of interest to investigate whether there is an interaction between APPs and $sPLA_2$. Of ten APPs tested, three were found to inhibit $sPLA_2$ activity. These included CRP, SAP and lactoferrin. CRP is one of the best studied acute phase proteins, increasing rapidly up to 1000-fold in APR.[34] Both $sPLA_2$ and CRP bind to the phospholipids of perturbed membranes of living cells[27] and to PC:Lyso PC substrate.[27,35] It was found that CRP is a strong inhibitor of $sPLA_2$ activity, acting most probably as a competitor for the substrate.[23] The substrates used in the former[23] and present study form vesicles of different phospholipid composition. It was found that binding of CRP to such vesicles is preferential when they are composed of PC:Lyso PC in the proportion of 2:1. Most probably it is related to optimally altered surface packing density of the substrate. PE:Lyso PE and PE:Lyso PC substrates were not susceptible to hydrolytic activity of $sPLA_2$.

Since CRP belongs to the family of pentraxins (PEP), two other pentraxins, SAP and limulin were also tested. The former was found to inhibit $sPLA_2$ whereas the latter did not. SAP is a major APP in mice[27] but a minor one in man,[36] increasing in APR only three-fold from the physiological level of 30–40 μg/ml to no more than 90 μg/ml.[27,36] It shares 60% homology in amino acid sequence with CRP.[27] SAP circulates in the form of two pentameric molecules bound 'face to face' and, in contrast to CRP, is glycosylated.[37] The gene for SAP is located on the long arm of chromosome 1 (1q12–1q23), close to the gene coding for CRP. It was suggested that both are products of an ancestral duplication event.[38] Whereas CRP binds mainly to phosphorylcholine,[36,39] SAP has high affinity to phosphorylethanolamine.[36] In our study, SAP inhibited $sPLA_2$ activity when either PE:Lyso PE or PC:Lyso PC were used as substrates; however, much lesser concentrations of SAP were needed to achieve $IC_{50}$ when PE:Lyso PE was employed. In human serum, SAP binds to $C_{4b}$-binding protein (C4BP) and to various types of phospholipid vesicles. These reactions were found to be calcium dependent and can be disrupted by phosphorylethanolamine. In our study phosphorylethanolamine did not block the inhibitory activity of SAP.

Limulin in *Limulus polyphemus* is analogous to CRP in humans. It shares only 25–30% amino acid homology with CRP[40,41] and SAP[41] and, similarly to CRP, binds avidly to phosphorylcholine.[34,42,43] Limulin shares with human CRP two regions of preserved residues, 52–67, respon-

**Table 2.** Acute phase proteins as inhibitors of $sPLA_2$

| Group | Inhibitors | Non-inhibitors |
|---|---|---|
| Pentraxins | CRP, SAP | Limulin |
| Iron binders | Lactoferrin | Transferrin |
| Bacteriostatic | Lactoferrin | Lysozyme |
| Amyloidogenic substances | SAP | SAA*, $\beta_2$-m |
| Negative APP | HDL* | BSA, HSA |
| Proteinase inhibitors | N/D$^+$ | $\alpha_2$-Macroglobulin |

*Published in *Biochem J* 1995; **309**: 461-464.
$^+$N/D: not done.

sible for the binding site to phosphorylcholine and 139–153 that binds $Ca^{2+}$.[41,44] The fact that, in contrast to human CRP, limulin does not inhibit $sPLA_2$ activity, may mean that either the above two binding sites are not responsible for inhibition of $sPLA_2$ activity, or that the affinity of limulin to the substrate is much weaker than that of CRP. Therefore, pentraxin structure does not automatically confer anti-$sPLA_2$ activity, since in contrast to CRP and SAP, limulin was not inhibitory. Neither amyloidogenic property of SAP can be linked to the inhibition of $sPLA_2$, since SAA[24] and $\beta_2$-microglobulin, both known participants in amyloidogenesis, did not inhibit $sPLA_2$ activity (Table 2).

Of three $sPLA_2$ inhibitors, lactoferrin was the most active. Lactoferrin is one of the iron-carrying proteins, produced mainly by polymorphonuclear cells.[45,46] Its synthesis is induced by TNF[45,46] and in sepsis it behaves like a classical acute phase protein[46,47] with the potential to increase in the circulation ten or more fold from its physiological level of 0.2–2.8 mg/ml.[46–48] Infusions of LPS to healthy volunteers[46] or to piglets,[48] or of *Escherichia coli* to piglets[47] lead to a marked increase in circulating lactoferrin. In turn, lactoferrin interacts with LPS, preventing iron-catalysed formation of hydroxyl radicals.[45] Lactoferrin protects mice against a lethal dose of *E. coli in vivo*,[47] acting as bacteriostatic glycoprotein and inducing damage to the outer membrane of Gram-negative bacteria.[49]

It seems, therefore, that lactoferrin acts upon Gram-negative bacteria similarly to bacterial permeability increasing protein (BPI).[2] Since $sPLA_2$ hydrolyses membrane phospholipids of microorganisms killed by BPI,[2] the fact that lactoferrin inhibits $sPLA_2$ activity may mean that it acts as a limiting factor in hydrolytic activity of the latter. The property of iron chelation by lactoferrin was not related to inhibitory anti-$sPLA_2$ activity, since transferrin, another iron chelator did not inhibit $sPLA_2$.

The fact that during the acute phase there is simultaneous co-induction and over-expression of both inhibitors such as CRP, SAP and lactoferrin, and enhancers such as SAA[24] of $sPLA_2$ activity, emphasizes the complexity of the organism's response to injury. Since, *in vivo*, various acute phase proteins are over-expressed to very different orders of magnitude, the end result regarding their impact on proinflammatory activity of $sPLA_2$ cannot be predicted.

### References

1. Dennis EA. Diversity of group types, regulation and function of phospholipase $A_2$. *J Biol Chem* 1994; **269**: 13057–13060.
2. Wright GW, Ooi CE, Weiss J, Elsbach P. Purification of a cellular (granulocyte) and an extracellular (serum) phospholipase $A_2$ that participate in the destruction of *Escherichia coli* in rabbit inflammatory exudate. *J Biol Chem* 1990; **265**: 6675–6681.
3. Harwig SSL, Tan L, Qu X-D, Cho Y, Eisenhauer PB, Lehrer RI. Bactericidal properties of murine intestinal phospholipase $A_2$. *J Clin Invest* 1995; **95**: 603–610.
4. MacPhee M, Chepenik KP, Liddell RA, Nelson KK, Siracusa LD, Buchberg AM. The secretory phospholipase $A_2$ gene is a candidate for the Mom1 locus, a major modifier of $Apc^{MIN}$-induced intestinal neoplasia. *Cell* 1995; **81**: 957–966.
5. Kennedy BP, Payette P, Mudgett J, *et al.* A natural disruption of the secretory group II phospholipase $A_2$ gene in inbred mouse strains. *J Biol Chem* 1995; **270**: 22378–22385.
6. Vadas P, Hay JB. Involvement of circulating phospholipase $A_2$ in the pathogenesis of the hemodynamic changes in endotoxin shock. *Can J Physiol Pharmacol* 1983; **61**: 561–566.
7. Vadas P, Pruzanski W. Induction of group II phospholipase $A_2$ expression and the pathogenesis of the sepsis syndrome. *Circulatory Shock* 1993; **39**: 160–167.
8. Vadas P, Pruzanski W, Stefanski E, *et al.* The pathogenesis of hypotension in septic shock: correlation of circulating phospholipase $A_2$ levels with circulatory collapse. *Crit Care Med* 1988; **16**: 1–7.
9. Vadas P, Schouten B, Stefanski E, Scott E, Pruzanski W. The association of hyperphospholipasemia $A_2$ with multisystem organ failure due to salicylate intoxication. *Crit Care Med* 1993; **21**: 1087–1091.
10. Vadas P, Pruzanski W, Stefanski E, *et al.* Phospholipase $A_2$ in acute bacterial peritonitis in man. In: Dennis EA, Hunter T, eds. *Cell Activation and Signal Initiation: receptor and phospholipase control of inositol phosphate, PAF, and eicosanoid production.* Alan R Liss, 1989; 311–316.
11. Pruzanski W, Koo Seen Lin M, Vadas P. Secretory phospholipase $A_2$ in rheumatic diseases. In: Glaser KB, Vadas P, eds. *Phospholipase $A_2$ in Clinical Inflammation. Molecular approaches to pathophysiology.* CRC Press, 1995; 127–147.
12. Pruzanski W, Vadas P, Fornasier V. Inflammatory effect of intradermal administration of soluble phospholipase $A_2$ in rabbits. *J Invest Dermatol* 1986; **86**: 380–383.
13. Cirino G, Cicala C, Sorrentino L, Maiello FM, Browning JL. Recombinant secreted nonpancreatic phospholipase $A_2$ induces a synovitis-like inflammation in the rat air pouch. *J Rheumatol* 1994; **21**: 824–829.
14. Vadas P, Pruzanski W, Kim J, Fornasier V. The proinflammatory effect of intra-articular injections of soluble human and venom phospholipase $A_2$. *Am J Pathol* 1989; **134**: 807–811.
15. Bomalaski JS, Clark MA. Phospholipase $A_2$ and arthritis. *Arth Rheum* 1993; **36**: 190–198.
16. Vadas P, Pruzanski W, Stefanski E, *et al.* Extracellular phospholipase $A_2$ secretion is a common effector pathway of interleukin-1 and tumour necrosis factor action. *Immunology Letters* 1991; **28**: 187–194.
17. Crowl RM, Stoller TJ, Conroy RR, Stoner CR. Induction of phospholipase $A_2$ gene expression in human hepatoma cells by mediators of the acute phase response. *J Biol Chem* 1991; **266**: 2647–2651.
18. Vadas P, Browning J, Edelson J, Pruzanski W. Extracellular phospholipase $A_2$ expression and inflammation: the relationship with associated disease states. *J Lipid Mediators* 1993; **8**: 1–30.
19. Goulding NJ, Guyre PM. Glucocorticoids, lipocortins and the immune response. *Current Opinion Immunol* 1993; **5**: 108–113.
20. Suwa Y, Kudo I, Imaizumi A, *et al.* Proteinaceous inhibitors of phospholipase $A_2$ purified from inflammatory sites in rats. *Proc Natl Acad Sci USA* 1990; **87**: 2395–2399.
21. Baumann H, Gauldie J. The acute phase response. *Immunol Today* 1994; **15**: 74–80.
22. Ramadori G, Sipe JD, Dinarello CA, Mizel SB, Colten HR. Pretranslational modulation of acute phase hepatic protein synthesis by murine recombinant interleukin-1 (IL-1) and purified human IL-1. *J Exp Med* 1985; **162**: 930–942.
23. Vadas P, Stefanski E, Grouix B, Schouten BD, Pruzanski W. Inhibition of human group II phospholipase $A_2$ by C-reactive protein *in vitro*. *J Lipid Mediat Cell Signalling* 1995; **11**: 187–200.
24. Pruzanski W, de Beer FC, de Beer MC, Stefanski E, Vadas P. Serum amyloid A protein enhances the activity of secretory non-pancreatic phospholipase $A_2$. *Biochem J* 1995; **309**: 461–464.
25. Steel DM, Whitehead AS. The major acute phase reactants. C-reactive protein, serum amyloid P component and serum amyloid A protein. *Immunol Today* 1994; **15**: 81–88.
26. Richards C, Gauldie J, Baumann H. Cytokine control of acute phase protein expression. *Eur Cytokine Net* 1991; **2**: 89–98.
27. Pepys MB, Baltz ML. Acute phase proteins with special reference to C-reactive protein and related proteins (pentaxins) and serum amyloid A protein. *Adv Immunol* 1983; **34**: 141–212.
28. Courtoy PJ, Lombart C, Feldmann G, Moguilevsky N, Rogier E. Synchronous increase of four acute phase proteins synthesized by the same hepatocytes during the inflammatory reaction. *Lab Invest* 1981; **44**: 105–115.
29. Heinrish PC, Castell JV, Andus T. Interleukin-6 and the acute phase response. *Biochem J* 1990; **265**: 621–636.
30. Richards CD, Shoyab M. The role of oncostatin M in the acute phase response. In: Mackiewicz A, Kushner I, Baumann H, eds. *Acute Phase Proteins: molecular biology, biochemistry, clinical applications.* Boca Raton: CRC Press, 1993; 321–327.
31. Franson R, Dobrow R, Weiss J, Elsbach P, Weglicki WB. Isolation and characterization of a phospholipase $A_2$ from an inflammatory exudate. *J Lipid Res* 1978; **19**: 18–23.
32. Vadas P, Wasi S, Movat HZ, Hay JB. A novel, vasoactive product and plasminogen activator from afferent lymph cells draining chronic inflammatory lesions. *Proc Soc Exp Biol Med* 1979; **161**: 82–85.
33. Vadas P, Taylor T, Molyneux M, Stefanski E, Pruzanski W. Serum phospholipase $A_2$ and disease severity in children with falciparum malaria. *Am J Trop Med Hyg* 1993; **49**: 455–459.
34. Schultz DR, Arnold PI. Properties of four acute phase proteins: C-reactive protein, serum amyloid A protein, $\alpha_1$-acid glycoprotein and fibrinogen. *Sem Arthr Rheum* 1990; **20**: 129–147.
35. Nagpurkar A, Saxena U, Mookerjea S. Interaction of rat serum phosphorylcholine-binding protein with phospholipid-containing liposomes. *J Biol Chem* 1983; **258**: 10518–10523.
36. Schwalbe RA, Dahlback B, Coe JE, Nelsestuen GL. Pentraxin family of proteins interact specifically with phosphorylcholine and/or phosphorylethanolamine. *Biochem* 1992; **31**: 4907–4915.
37. Swanson SJ, Christner RB, Mortensen RF. Human serum amyloid P-component (SAP) selectively binds to immobilized or bound forms of C-reactive protein (CRP). *Biochim Biophys Acta* 1992; **1160**: 309–316.
38. Dowton SB, Colten HR. Acute phase reactants in inflammation and infection. *Sem Hematol* 1988; **25**: 84–90.
39. Volanakis JE, Xu Y, Macon KJ. Human C-reactive protein and host defence. In: Marchalonis J, Reinisch C, eds. *Defense Molecules. (UCLA Symposia on Molecular and Cellular Biology. New Series).* Alan R. Liss, 1990; **121**: 161–175.
40. Ying S-C, Marchalonis JJ, Gewurz AT, *et al.* Reactivity of anti-human C-reactive protein (CRP) and serum amyloid P component (SAP) monoclonal antibodies with limulin and pentraxins of other species. *Immunol* 1992; **76**: 324–330.
41. Vasta GR. Invertebrate lectins, C-reactive proteins and serum amyloid. Structural relationships and evolution. In: Marchalonis J, Reinisch C, eds. *Defense Molecules. (UCLA Symposia on Molecular and Cellular Biology. New Series).* Alan R. Liss, 1990; **121**: 183–199.
42. Tennent GA, Butler PJG, Hutton T, *et al.* Molecular characterization of *Limulus polyphemus* C-reactive protein. I. Subunit composition. *Eur J Biochem* 1993; **214**: 91–97.
43. Robey FA, Liu T-Y. Limulin: a C-reactive protein from *Limulus polyphemus. J Biol Chem* 1981; **256**: 969–975.
44. Nguyen NY, Suzuki A, Cheng SM, Zon G, Liu TY. Isolation and characterization of *Limulus* C-reactive protein genes. *J Biol Chem* 1986; **261**: 10450–10455.
45. Cohen MS, Mao J, Rasmussen GT, Serody JS, Britigan BE. Interaction of lactoferrin and lipopolysaccharide (LPS): effects on the antioxidant property of lactoferrin and the ability of LPS to prime human neutrophils for enhanced superoxide formation. *J Infec Dis* 1992; **166**: 1375–1378.
46. Nuijens JH, Abbink JJ, Wachtfogel YT, *et al.* Plasma elastase alpha 1-antitrypsin and lactoferrin in sepsis: evidence for neutrophils as mediators in fatal sepsis. *J Lab Clin Med* 1992; **119**: 159–168.
47. Zagulski T, Lipinski P, Zagulska A, Broniek S, Jarzabek Z. Lactoferrin can protect mice against a lethal dose of *Escherichia coli* in experimental infection *in vivo. Br J Exp Path* 1989; **70**: 697–704.
48. Gutteberg TJ, Rokke O, Andersen O, Jorgensen T. Early fall of circulating iron and rapid rise of lactoferrin in septicemia and endotoxemia: an early defence mechanism. *Scand J Infect Dis* 1989; **21**: 709–715.
49. Ellison III RT, Giehl TJ. Killing of Gram-negative bacteria by lactoferrin and lysozyme. *J Clin Invest* 1991; **88**: 1080–1091.

**Received 24 January 1996; accepted 22 February 1996**
